# A mouse model for studying chronic *Salmonella* Typhi infection and anti-biofilm interventions

**DOI:** 10.1128/mbio.03476-25

**Published:** 2025-12-18

**Authors:** Allysa L. Cole, Katherine J. Woolard, Amy Sorge, Christian Melander, John S. Gunn

**Affiliations:** 1 Center for Microbe and Immunity Research, Abigail Wexner Research Institute at Nationwide Children's Hospital 51711, Columbus, Ohio, USA; 2 Infectious Diseases Institute, The Ohio State University 2647, Columbus, Ohio, USA; 3 Department of Veterinary Biosciences, The Ohio State University 2647, Columbus, Ohio, USA; 4 Department of Chemistry and Biochemistry, University of Notre Dame 6111, Notre Dame, Indiana, USA; 5 Department of Pediatrics, College of Medicine, The Ohio State University 2647, Columbus, Ohio, USA; University of Illinois Chicago, Chicago, Illinois, USA

**Keywords:** *Salmonella*, collaborative cross, biofilms, chronic typhoid fever

## Abstract

Typhoid fever, caused by *Salmonella enterica* serovar Typhi (*S*. Typhi), continues to cause significant human disease, especially in endemic regions. Chronic carriers of typhoid fever are a significant contributor to the perpetuation of disease in these communities, and treatments for elimination of the carrier state remain a challenge to clinicians and researchers. *S*. Typhi is host-restricted to humans and has been shown to be rapidly cleared by immunocompetent mice, which led to the use of *S. enterica* serovar Typhimurium infection of susceptible mice as a model for *S*. Typhi and typhoid fever. Thus, the lack of a direct murine model of *S*. Typhi infection has long been a challenge in the study of the mechanisms of typhoid fever and identification of therapeutics to treat the chronic carrier state. Here, we demonstrate the establishment of a murine model of chronic *S*. Typhi infection utilizing the immunocompetent Collaborative Cross mouse lines CC003/Unc and CC053/Unc, in which *S*. Typhi can be recovered in the gallbladder, liver, and spleen up to 21 days post-infection. Cholesterol gallstones foster enhanced *S*. Typhi gallbladder carriage. While there is no sex difference with regard to CFU recovered in the gallbladder at 21 days post-infection, male mice lost significantly more weight at early time points after infection and showed increased lethality. Bacterial aggregates (biofilms) were identified associated with the gallstone surface, consistent with observations in the *S*. Typhimurium gallstone mouse model of carriage and in human *S*. Typhi carriers. Additionally, we show that novel anti-biofilm compounds, in combination with ciprofloxacin, are able to reduce *S*. Typhi burden within the CC003/Unc gallbladder. Together, these findings support the role of gallstone biofilms in chronic typhoid fever and establish a new murine model that can be used to further interrogate the mechanisms of chronic typhoid fever utilizing the primary etiologic agent, *S*. Typhi.

**IMPORTANCE:**

Chronic typhoid fever, caused by persistent *Salmonella* Typhi infection, remains a significant public health concern in multiple regions throughout the world. There is currently no direct animal model utilizing *S*. Typhi that has been demonstrated to recapitulate the carrier state of typhoid fever. This lack of an animal model has precluded *in vivo* studies on the mechanisms of infection unique to this serovar. This study establishes and characterizes a new murine model of chronic *S*. Typhi carriage and demonstrates its utility with the identification of novel anti-biofilm compounds that disperse *S*. Typhi biofilms from gallbladder gallstones. This new model will provide a means for further studies into *S*. Typhi chronic infection.

## INTRODUCTION

*Salmonella enterica* serovar Typhi (*S*. Typhi) is the causative agent of typhoid fever, a systemic, febrile illness in humans with over 10 million cases and 148,000–161,000 deaths worldwide (1). Following ingestion of contaminated food or water, the bacteria, through multiple mechanisms, penetrate the intestinal epithelial layer, proliferate, and spread systemically causing acute disease (2). Though typhoid fever has been controlled through improved sewage and waste handling in areas like North America, Europe, and Australia, it remains endemic in multiple developing regions, such as Sub-Saharan Africa and Southeast Asia, posing an important health risk for these populations and those traveling to these regions (3). While antimicrobial use remains a mainstay of treatment and control of acute infection, antibiotic resistance is increasing in prevalence, prompting the World Health Organization in 2018 to identify *S*. Typhi as a high-priority target for new antibiotic strategies (4, 5).

While most patients are able to clear acute *S*. Typhi infection through treatment, approximately 2%–5% of individuals become asymptomatic, chronic carriers (6). In the chronic carrier state, bacteria are resistant to immune clearance and antimicrobials and can continue to intermittently spread through the individual’s feces and urine (7). This chronic carriage plays an integral role in the cycle of *S*. Typhi infection, as there are no known disease vectors other than humans (8, 9). The gallbladder has been identified as a primary site of persistent infection through formation of *Salmonella* biofilms on gallstones (7, 10, 11). Biofilms are microbial communities within a complex matrix of self-produced extracellular substances which aid in surface attachment while simultaneously providing protection from immune clearance and other environmental stressors (12, 13). The biofilm state contributes to significant resistance to antibiotics, with up to a 1,000-fold increase in resistance in comparison to bacteria in their free-floating, planktonic state (12–15). Given these protections, it is not surprising that antibiotic use is ineffective for the treatment of chronic *S*. Typhi carriers, as the most effective strategies currently in patients with gallstones focus on removal of the gallbladder and associated biofilms in combination with antibiotics (16, 17).

As *S*. Typhi is host-restricted to humans, *in vivo* studies of typhoid fever pathogenesis have primarily utilized susceptible mouse models infected with *S. enterica* serovar Typhimurium (*S*. Typhimurium). Mice infected with *S*. Typhimurium develop a systemic disease similar to that seen in human cases of typhoid fever (18). Our lab has utilized a mouse model that recapitulates the human carrier state of typhoid fever through use of *S*. Typhimurium infection of mice with gallstones, where the bacteria form biofilms on cholesterol gallstones (10). Using this model, we have previously shown the efficacy of anti-biofilm compounds on inhibition and dispersion of *Salmonella* biofilms (19). However, evaluating the effect of these compounds on *S*. Typhi has been precluded by the lack of a murine *S*. Typhi model, as common lab strains of mice quickly clear the infection when challenged with *S*. Typhi unless they are humanized or immunocompromised (20–22). While immunocompromised mice can be infected with *S*. Typhi, these models are unable to be used to properly assess host response to infection due to their undeveloped immune system (20, 21).

Recently, the Collaborative Cross project developed a large panel of recombinant inbred mouse strains with the goal of increasing the genetic variability in mice to research complex traits, which have been utilized for studies on infectious diseases (23, 24). Screening of a subset of these lines identified two strains, CC003/Unc and CC053/Unc, which are immunocompetent and permissive to acute *S*. Typhi infection (25). Interestingly, these two lines are not deficient in *Slc11a1* nor *Ncf2*, which are two genes that have been shown to affect *Salmonella* susceptibility in multiple laboratory mouse strains (26, 27). While this study demonstrated that *S*. Typhi could be recovered 6 days after infection within the liver and spleen, there was no evaluation of how long the infection can persist in the model (25). Notably, the presence or absence of *S*. Typhi was not evaluated in the gallbladder, raising the question of whether these strains could model long-term carriage within the gallbladder similar to the established *S*. Typhimurium model.

In this study, we examined the CC003/Unc and CC053/Unc mouse strains for their permissiveness to chronic *S*. Typhi infection by combining it with our established gallbladder carriage model. Upon successful model development, we report the efficacy of novel anti-biofilm compounds on *S*. Typhi biofilms both *in vitro* and *in vivo*. The murine *S*. Typhi chronic carriage model described here will provide an important tool for interrogating microbe-host interactions as well as assessing new therapeutic options for clearance of the chronic carrier state.

## RESULTS

### CC003/Unc and CC053/Unc mice are permissive to chronic *S*. Typhi infection

**Fig 1:**
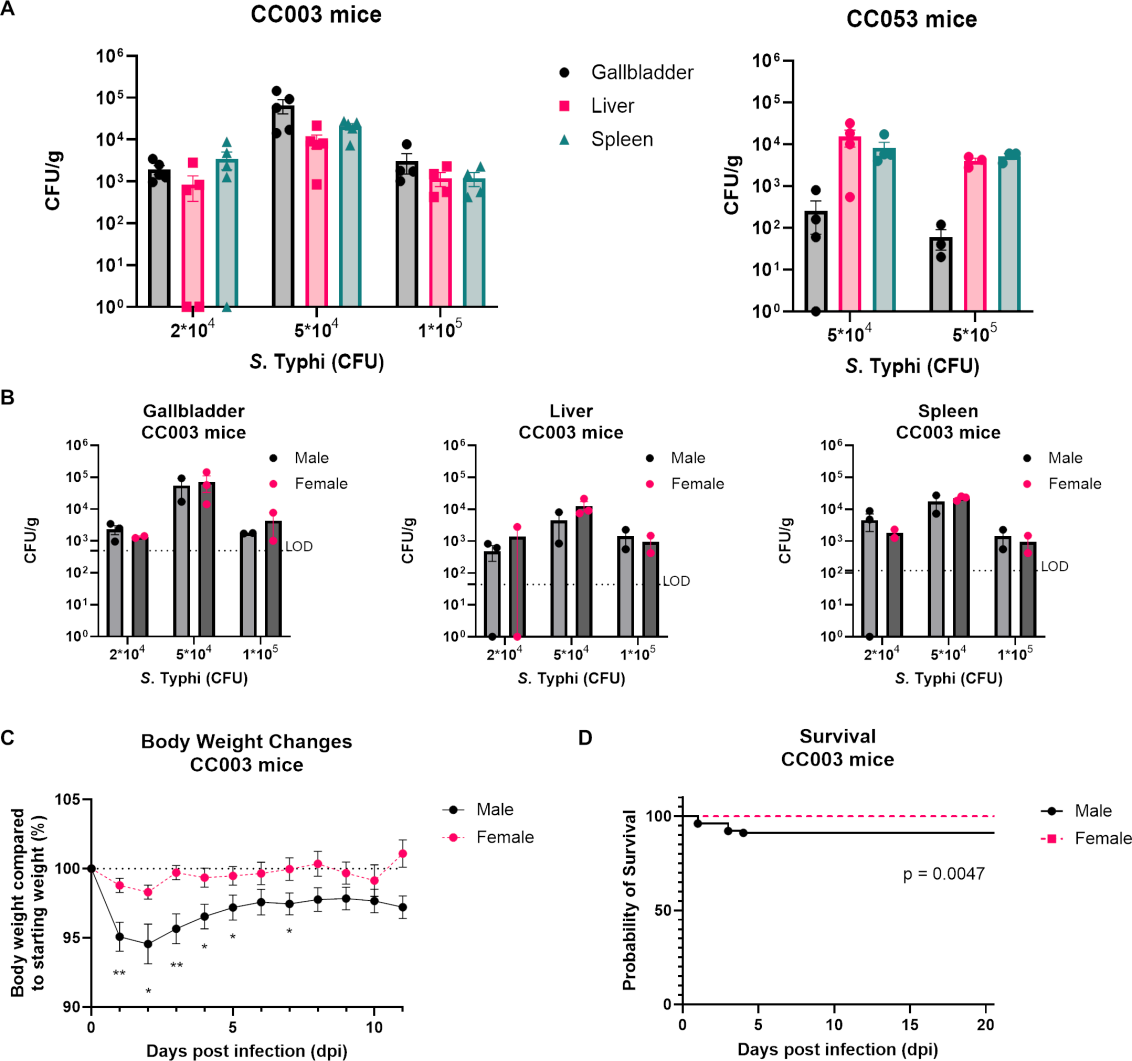
*S*. Typhi infection in CC003/Unc and CC053/Unc mice. Mice were fed a lithogenic diet for 6 weeks before transitioning to normal diet. After returning to normal diet, mice were IP infected with 2 × 10^4^ to 5 × 10^5^ CFUs of *S*. Typhi. (**A**) *S*. Typhi CFUs/gram from the gallbladder, liver, and spleen at 21 dpi in CC003/Unc and CC053/Unc mice at each inoculum tested. (**B**) *S*. Typhi CFUs/gram from each organ stratified by mouse sex in the CC003/Unc mice. Males and females were compared by Mann-Whitney test with no statistical significance found. Data represent geometric mean with standard error from the mean. LOD: limit of detection. (**C**) CC003/Unc mice were IP infected with 2 × 10^5^ CFUs of *S*. Typhi. Mice (*n* = 16 female, 15 male) were weighed daily beginning on the day of infection, and weights were compared to starting weight at 0 dpi. One-sample *t*-test with a hypothetical mean of 0 was performed. ** = *P*-value: < 0.01; * = *P*-value: <0.05. (**D**) Kaplan-Meier survival curve of all CC003/Unc mice used in multiple experiments infected with 2 × 10^4^ to 2 × 10^5^ CFU of *S*. Typhi. *n* = 95 female and 110 male mice.

A recent study showed that *S*. Typhi could be recovered from the liver and spleen of CC003/Unc and CC053/Unc mice at 6 days post-infection (dpi) (25). We sought to determine whether the CC003/Unc or CC053/Unc mice were able to be chronically infected via gallstone biofilms with *S*. Typhi. To do so, we fed CC003/Unc and CC053/Unc mice a lithogenic diet to induce gallstone formation before intraperitoneal (IP) infection with *rpoS^+^* wild-type (WT) *S*. Typhi (JSG4383). We initially sought to identify an inoculating dose that could consistently establish infection while being as close to a physiologically relevant dose as possible. At all concentrations of *S*. Typhi inocula tested (2 × 10^4^ to 5 × 10^5^ CFUs), we were able to recover *S*. Typhi in the gallbladder, liver, and spleen at 21 dpi in both mouse lines, and there was no clear dose response pattern in the amount of recoverable CFUs (Fig. 1A and B). While there was a significant increase in recovered bacteria in the gallbladder and liver of CC003 mice between the 2 × 10^4^ and 5 × 10^4^ CFU inoculum groups (*P*-values of 0.0155 and 0.0375, respectively; Kruskal-Wallis test), this significant increase was no longer observed when comparing the 2 × 10^4^ and 1 × 10^5^ CFU groups. The time point of 21 dpi was chosen because this is where the immune response to *Salmonella* infection in the gallbladder has been shown to shift from a Th1 pro-inflammatory to Th2 anti-inflammatory state, consistent with chronic infection (28). There were no significant observable differences between the CC003/Unc and CC053/Unc mice, and CC053/Unc mice were difficult to breed, so further experiments used only the CC003/Unc line.

Because Alugupalli et al. (25) reported sex differences in liver colonization at 6 dpi, we examined the organ burden in infected males and females at 21 dpi. We observed that male and female CC003/Unc mice had similar CFU within the gallbladder, liver, and spleen (Fig. 1B). Given this lack of a differential effect between the sexes, we have continued to use both male and female mice in our experiments, and no significant difference has arisen in subsequent experiments. However, male mice showed increased morbidity and mortality during the first few days of infection, with up to 10% of male mice succumbing to infection, and had significantly more weight loss than females within the first 72 hours (Fig. 1C and D).

During early experiments for the establishment of the model, we tested mice that were fed a lithogenic diet for either 6 or 8 weeks for gallstone formation. Mice fed the lithogenic diet for a longer duration had significantly increased liver weights, which decreased over time after return to normal diet (Fig. S1A and B). These weight increases in mice fed a lithogenic diet corresponded with lipid accumulation and lipid-granuloma formation in the liver on histologic evaluation (Fig. S1C), and these changes were present in the livers of mice fed a lithogenic diet but not infected with *S*. Typhi (Fig. S1D). Following these observations, we adjusted the protocol to only feed the lithogenic diet for 6 weeks and allowed for up to 1 week acclimation back to normal diet before infection. This significantly decreased the observed histologic changes at 21 dpi, and we were able to visualize characteristic foci of necrosis within infected livers (Fig. S1E). In our experience, these histologic changes secondary to the lithogenic diet are not as pronounced in the *S*. Typhimurium chronic carriage model using the 129X1/SvJ mouse line.

### *S*. Typhi aggregates are associated with cholesterol gallstones and gallstone formation increases chronic *Salmonella* burden in the gallbladder

**Fig 2:**
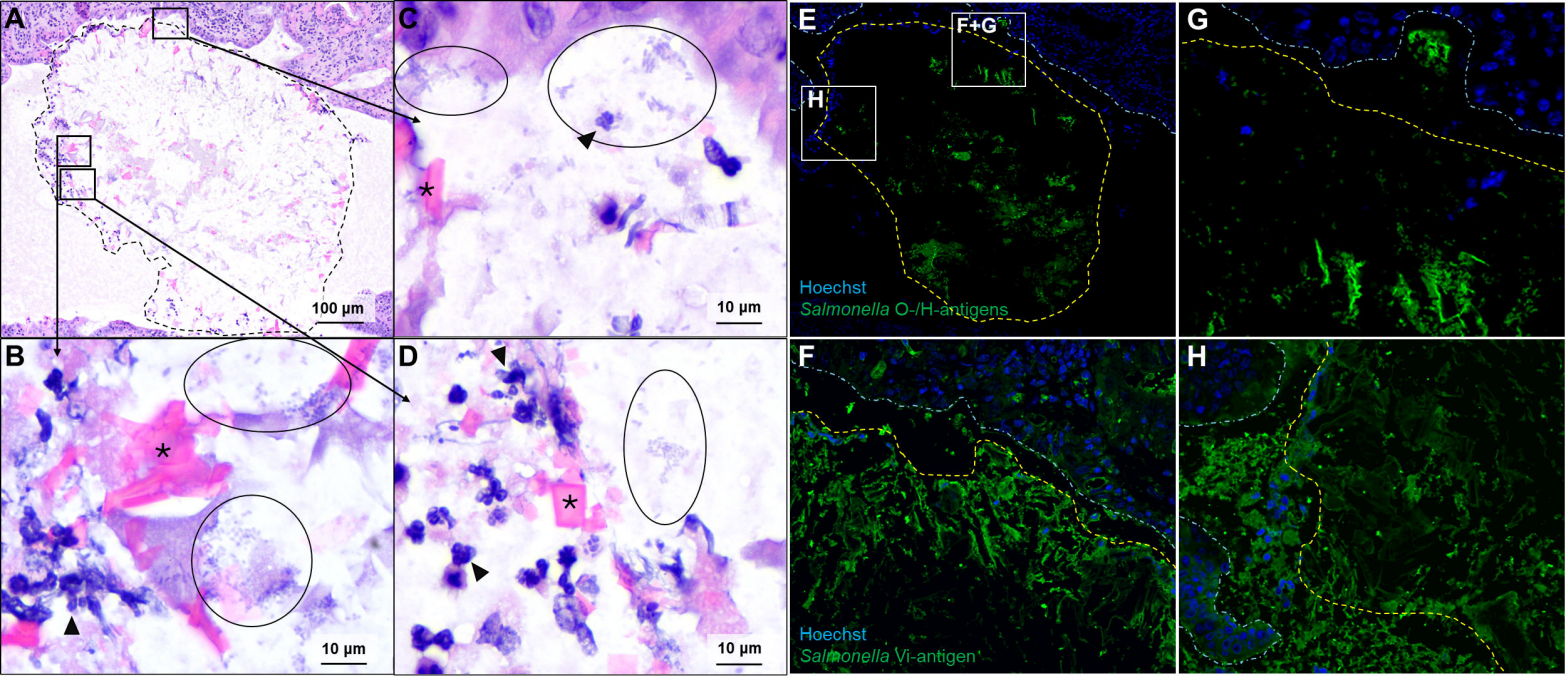
*S*. Typhi bacterial aggregates are closely associated with gallstones. (**A**) Representative image of a gallstone (dashed outline) within the gallbladder lumen. 10×. H&E. (**B–D**) Magnified fields from panel A show aggregates of short rod bacteria (circled) mixed with occasional degenerate neutrophils (arrowhead) near the surface of the gallstone and adjacent to mucosal epithelium. Crystalline structures (*) are present throughout the gallstone. 100×. H&E. (**E-H**) Immunofluorescence imaging of gallstone and bacteria. The gallstone surface is indicated by a dashed yellow line, and the mucosal epithelium surface is indicated by a dashed white line. Epithelial cell nuclei are labeled blue with Hoescht, and *Salmonella* O- and H-antigens (**E and G**) and Vi-antigen (**F and H**) that label immunopositive are green.

**Fig 3:**
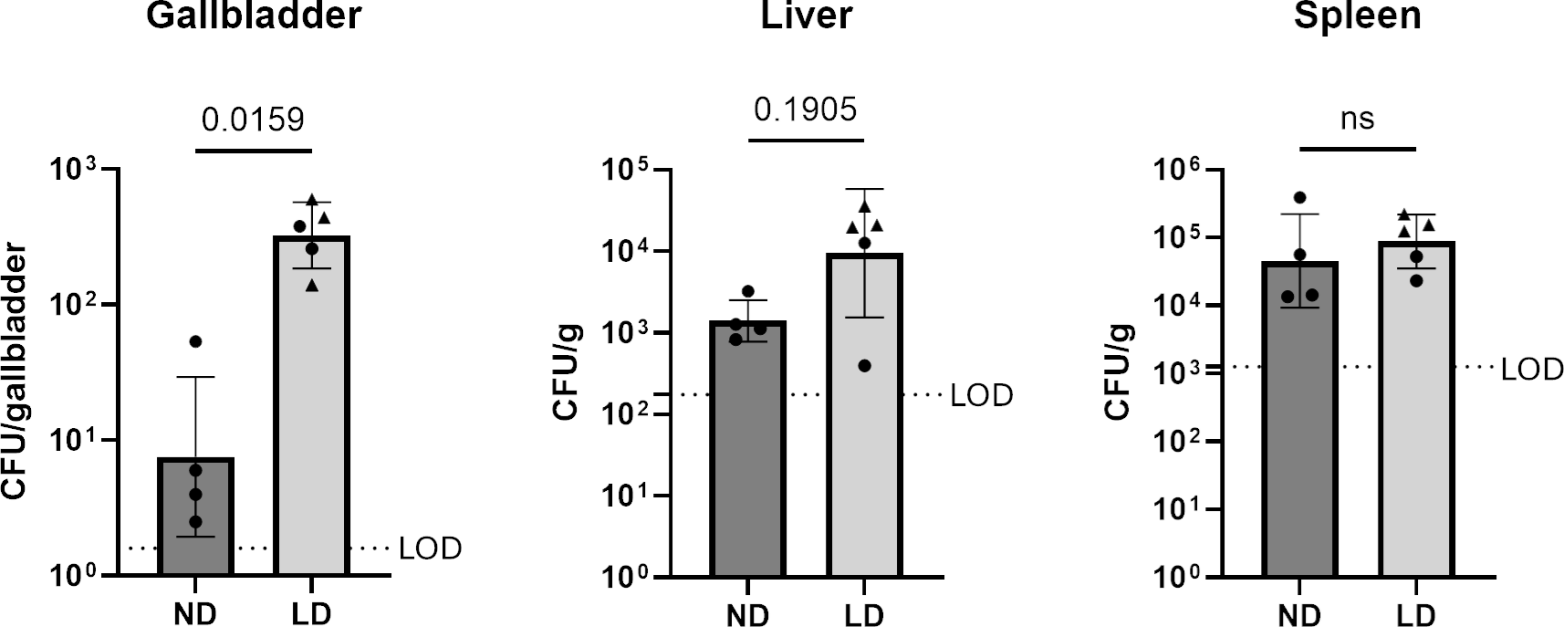
Effect of lithogenic diet on chronic *Salmonella* burden. CC003/Unc mice were fed either normal diet or a lithogenic diet for 6 weeks. All mice were infected with 5 × 10^4^ CFUs of *S*. Typhi IP, and tissues were collected 21 dpi. ND = mice on normal diet (*N* = 4); LD = mice on lithogenic diet (*n* = 5). Males are represented by circles and females are triangles. Data represent the geometric mean with a standard error of the mean (SEM) of CFU per whole gallbladder or CFU per gram of tissue. ND and LD are compared by Mann-Whitney test. ns = not significant. LOD: limit of detection.

It has been established that *S*. Typhimurium and *S*. Typhi form bacterial biofilms on murine and human gallstones, respectively. Gallstone biofilms have been shown to be a significant contributor to chronic carriage (10). In order to interrogate the role of biofilms within our mouse model, we first needed to demonstrate that *S*. Typhi forms biofilms on the gallstones within their gallbladders. Gallstones were grossly visible in the gallbladder during tissue collection, and these gallstones can be seen histologically on routine hematoxylin and eosin (H&E) staining within the lumen of the gallbladder (Fig. 2A). Numerous aggregates of short rod bacteria, morphologically consistent with *Salmonella*, are observed associated with the surface of the gallstone, and these bacteria are occasionally accompanied by neutrophils and other cellular debris (Fig. 2B through D). These bacteria are immunopositive for antibodies against *Salmonella*-specific lipopolysaccharide (LPS) and flagellar proteins (Fig. 2E and G) as well as *S*. Typhi Vi-antigen (Fig. 2F and H). Together, these findings support the presence of *S*. Typhi biofilms associated with gallstones within infected CC003/Unc mice. To assess the role of these gallstone-associated biofilms and their contribution to chronic carriage, we compared mice that were fed either a normal diet or lithogenic diet. Mice fed a lithogenic diet have a significantly higher *S*. Typhi burden in their gallbladder, with no significant differences in the spleen or liver (Fig. 3).

### Anti-biofilm compounds can inhibit and disperse *S*. Typhi biofilms *in vitro* and *in vivo*

Our group has previously demonstrated the efficacy of the anti-biofilm compound JG-1 on inhibition and dispersion of *Salmonella* biofilms both *in vitro* alone and *in vivo* in conjunction with ciprofloxacin in a mouse model utilizing *S*. Typhimurium (19). JG-1 has undergone significant derivatization in an effort to improve its activity (29). While this approach generated compounds with improved activity, the potential for undesirable pre-clinical characteristics still existed.

**Fig 4:**
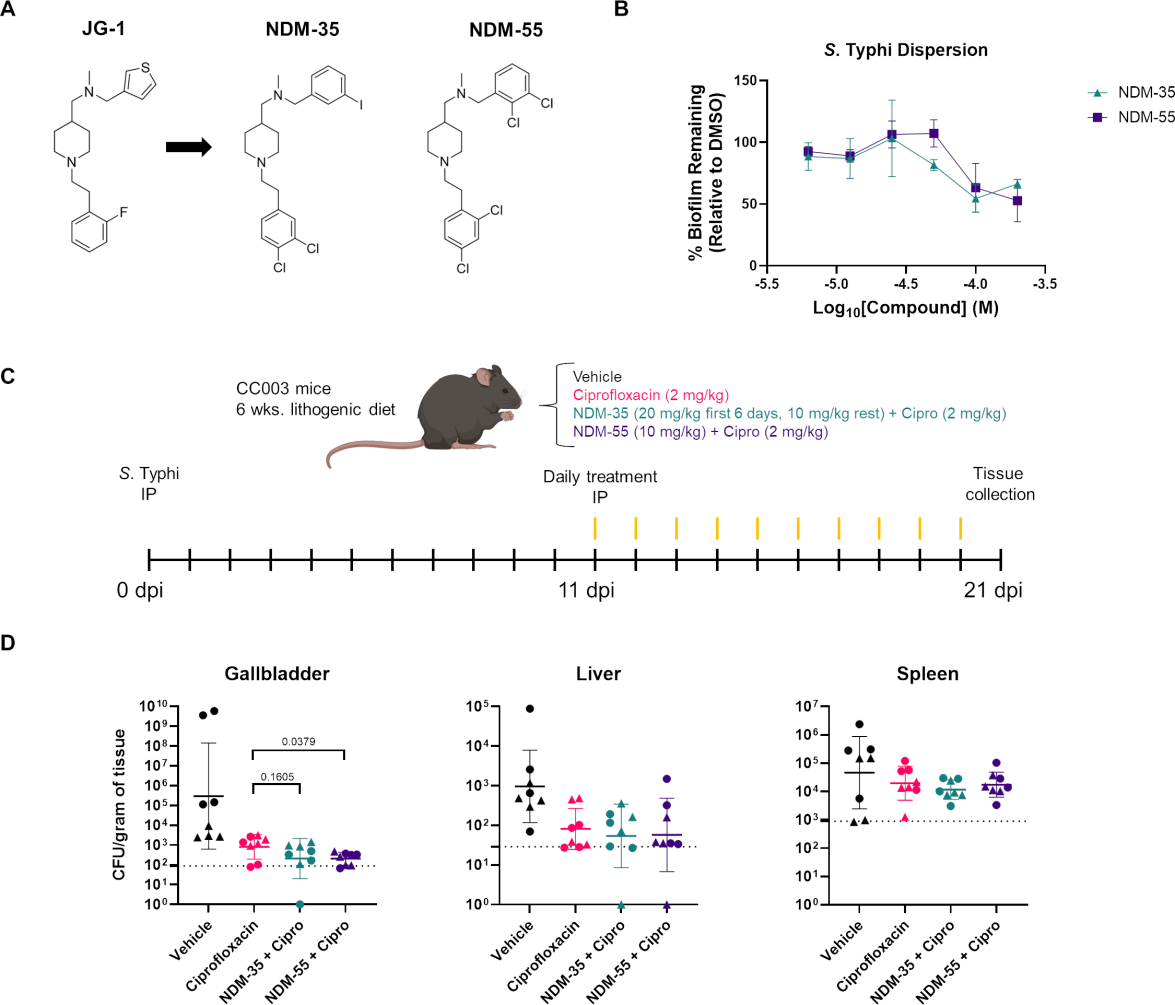
Dispersion of *S*. Typhi biofilms using novel small-molecule anti-biofilm compounds. (**A**) Molecular structure of parent compound JG-1 and derivatives NDM-35 and NDM-55. (**B**) Dispersion activity of NDM-35 and NDM-55 on 4-day-old *S.* Typhi biofilms. (**C**) Graphical outline of infection and treatment of CC003/Unc mice with anti-biofilm compounds in conjunction with ciprofloxacin. CC003/Unc mice were fed a lithogenic diet for 6 weeks prior to infection. All mice were infected with 2 × 10^5^ CFU of *S.* Typhi IP. Mice were treated daily with assigned compounds IP beginning 11 dpi (*N* = 8 per group). (**D**) Individual organ CFU of treated mice. Males are represented by circles and females are triangles. Data represent the geometric mean with a standard error of the mean (SEM) of CFU per whole gallbladder or CFU per gram of tissue. Groups are compared by Mann-Whitney test. LOD: limit of detection.

Through testing of newly generated JG-1 derivatives on *S*. Typhimurium biofilms (Fig. S2; the complete medicinal chemistry and identification of these lead molecules will be described in a parallel manuscript), we identified seven lead compounds to further test on *S*. Typhi biofilms. Applying these compounds to pre-established *S*. Typhi biofilms in cholesterol-coated 96-well plates showed six compounds performing better biofilm dispersion than JG-1, which poorly dispersed *S*. Typhi biofilms, with EC_50_s ranging from 191.4 μM to 593.4 μM. We identified NDM-35 and NDM-55 as the best candidates to pursue as possible therapeutic compounds (Fig. 4A). Both NDM-35 and NDM-55 were able to disperse 96-hour-old biofilms, with a calculated EC_50_ of 328.6 μM and 191.4 μM, respectively. Neither NDM-35 nor NDM-55 has direct bactericidal effects on *S*. Typhi, highlighting that the observed results are due to the effect of the compounds on the bacterial biofilms rather than killing of the bacteria (data not shown).

Next, as proof of principle for the compounds and the mouse model, we tested the efficacy of NDM-35 and NDM-55 on a chronic *S*. Typhi carriage by treating *S*. Typhi infected CC003/Unc mice that were previously fed a lithogenic diet. Following *S*. Typhi infection, we waited 11 days to allow for the establishment of gallbladder infection and biofilm formation on gallstones before beginning treatment with daily IP injections of vehicle, ciprofloxacin alone, or ciprofloxacin in conjunction with NDM-35 or NDM-55. After 10 days of treatment (21 dpi), tissue collection and bacterial quantification were performed (Fig. 4C). Ciprofloxacin was given to kill the bacteria dispersed from the biofilm by the compounds, and previous work with the parent compound JG-1 in the 129X1/SvJ model showed a significant increase in systemic *S*. Typhimurium and mouse mortality when the compound was given alone (19). A loading dose (20 mg/kg/day) of NDM-35 was given for the first 6 days of treatment before being treated with a lower dose (10 mg/kg/day) for the remaining days. NDM-55 was given at 10 mg/kg/day for the entirety of the treatment period due to limitations in solubility. While there was a significant decrease in CFUs in ciprofloxacin-only treated compared to vehicle-treated mice in the gallbladder (*P* = 0.019) and liver (*P* = 0.0112), treatment with a combination of ciprofloxacin and compounds resulted in a further decrease in gallbladder CFUs, with a significance observed between NDM-55 + ciprofloxacin and ciprofloxacin alone (Fig. 4D). No significant difference was observed between the compound + ciprofloxacin groups and ciprofloxacin alone in either the liver or spleen.

## DISCUSSION

Typhoid fever research has long lacked a direct *in vivo* model due to the host specificity of *S*. Typhi, as most immunocompetent, non-humanized, and commonly used inbred research mouse lines rapidly clear *S*. Typhi infection, leading to the extensive use of *S.* Typhimurium infection of susceptible mice as a primary model of typhoid fever in humans. However, recently the Collaborative Cross strains CC003/Unc and CC0053/Unc were shown to be permissive to acute *S*. Typhi infection up to 6 dpi, have an intact immune system, and are able to mount *S*. Typhi-specific immune response (25). Building on this study, we sought to validate these findings and to use these mice to attempt to establish a new murine model of chronic typhoid fever carriage using *S*. Typhi as the infectious agent in order to more accurately model chronic typhoid fever in humans. As biofilm formation on gallstones is a well-established mechanism of chronic carriage, we fed a lithogenic diet to the CC053/Unc and CC003/Unc mouse lines to provide a substrate for biofilm formation (7–11). We show that this new model consistently carries *S*. Typhi infection within the gallbladder, liver, and spleen up to 21 dpi, and the presence of gallstones contributes to a significantly increased bacterial burden within the gallbladder. Aggregates of *S*. Typhi can be seen closely associated with these gallstones on histology, supporting the role of biofilms on chronic carriage within our model.

Previously used models of *S*. Typhi induced typhoid fever have focused on immunodeficient or humanized mice (20, 21, 30, 31) as well as non-human primates, such as chimpanzees, where it can cause transient bacteremia and limited disease (32–34). However, the pathology is usually milder and not fully representative of human typhoid fever.

Additionally, mice deficient in CMP-*N*-acetyl neuraminic acid hydroxylase (CMAH) have also been used as a model of *S*. Typhi infection (35). It was previously shown that mice deficient in CMAH are unable to convert *N*-acetyl neuraminic acid (Neu5Ac) to *N*-glycolylneuraminic acid (Neu5Gc) (36). Unlike most mammals, humans naturally lack CMAH (37), causing the surface glycoproteins of cells to have significantly more terminal Neu5Ac residues, which have been demonstrated to be a strong binding target of the *Salmonella* typhoid toxin expressed by *S*. Typhi and *S*. Paratyphi (38, 39). While Song et al. (35) were able to recover *S*. Typhi (ISP2825) from the liver and spleen 3 dpi in the CMAH-null mice, their WT strain was rarely recovered from the gallbladder at this time point. These features may limit the use of this model for studies of persistent *S*. Typhi infection, though more studies are necessary to further characterize this mouse line at later time points.

Our group has previously shown the efficacy of small-molecule anti-biofilm compounds, including JG-1, on dispersion of various bacterial biofilms including *Salmonella* through complex mechanisms (19, 29, 40, 41). These compounds offer a novel therapeutic option to treat biofilm-mediated infections, which are estimated in the United States to underlie 17 million infections causing over 550,000 deaths each year (42, 43). The new mouse model described here provides a powerful tool to continue screening and investigating these compounds for treatment of the chronic carrier state of human typhoid fever. NDM-35 and NDM-55 are new JG-1 derivatives with increased efficacy against *in vitro S.* Typhimurium biofilms. Both showed decreased recoverable bacteria in the gallbladder of CC003/Unc mice upon delivery post-infection, highlighting the efficacy in removing *S*. Typhi from the organ associated with chronic carriage and biofilm formation. Interestingly, we saw no significant effect on CFUs in the livers of mice given the compounds paired with ciprofloxacin when compared with vehicle-treated mice, while ciprofloxacin alone showed a significant decrease in hepatic CFUs. Given the lack of direct bactericidal activity of these compounds on *S*. Typhi, these findings are consistent with the hypothesis that these compounds act more directly on biofilms, which are more heavily involved in gallbladder colonization, than the bacteria themselves. However, the intracellular lifecycle of *Salmonella* within the liver and spleen cannot be discounted as a possible contributor to these differences, and further studies on these compounds are necessary. Additionally, ciprofloxacin alone had a significant impact on the CFUs within the gallbladder and spleen, which may be masking the effect of the tested compounds. Additional testing of these compounds with a lower concentration of ciprofloxacin may allow for a more clear effect.

While there is no difference in bacterial CFUs in any of the organs evaluated at 21 dpi between male and female CC003/Unc mice, we noted a significant increase in weight loss and mortality within the acute phase of infection in male mice. Alugupalli et al. (25) noted an increased amount of *S*. Typhi within the spleens of male CC003/Unc mice at 6 dpi, which may correlate with the morbidity and mortality that we have observed in our model. In human typhoid fever, it has been demonstrated that males are more likely to develop more serious clinical disease and are at a higher risk for intestinal perforation and subsequent mortality (44–48). Additionally, sexual dimorphism in immune response to infection is well-established across many organ systems, though there are few studies on this subject in regard to *Salmonella* infections (45, 49–53). The clinical differences we observed in the CC003/Unc mouse model suggest a difference in host immune response during the acute phase of infection, and further studies using this model may aid in better mechanistic understanding of the effect of hormones in the systemic immune response during *Salmonella* infection.

Interestingly, in mice fed a lithogenic diet, we noted a trend toward an increase in *S*. Typhi within the liver, which is intimately associated with the gallbladder. Currently, the mechanism of this increase in bacterial burden is unclear. While bile typically flows from the gallbladder into the gastrointestinal tract, it is known that gallstones can cause cholestasis and backup of bile into the liver among other hepatic changes. Bile duct ligation resulting in cholestasis and liver fibrosis in mouse models has been shown to cause immunologic changes in the liver, particularly increased interleukin (IL)-10 release following bacterial infection and decreased bacterial immunity (54). Given the known role of IL-10 in promoting systemic *Salmonella* infection, these changes could further aid *S*. Typhi in long-term colonization of the liver in the lithogenic-diet fed mice (55, 56).

*S.* Typhi naturally infects its hosts through the oral-fecal route. IP infection is used in this model as the initial experiments did not observe permissiveness of oral *S*. Typhi in the CC003 (25). This is a drawback of the model, as it relies on systemic infection through a less-natural route. However, the IP route establishes consistent infection of the gallbladder, making this model well suited for evaluating the gallbladder environment and the chronic carrier state of typhoid fever. With this model, additional studies on the mechanisms and virulence factors of *S*. Typhi pathogenesis; preventative measures, such as Vi-polysaccharide conjugate vaccines and their efficacy; and novel therapeutics can be performed.

## MATERIALS AND METHODS

### Mice

Mouse care and housing were carried out in accordance with guidelines established by the Abigail Wexner Research Institute (AWRI) Institutional Animal Care and Use Committee (IACUC). The AWRI IACUC is accredited by the Association for the Assessment and Accreditation of Laboratory Animal Care International. All research activities adhered to the statutes of the Animal Welfare Act and the guidelines of the Public Health Service as required in the *Guide for the Care and Use of Laboratory Animals*. Mice were obtained from the Systems Genetics Core Facility, University of North Carolina at Chapel Hill (Chapel Hill, NC, USA) and maintained and bred in a specific-pathogen-free facility in AWRI.

### Bacterial strains

The bacterial strain used in this study is *S.* Typhi Ty2 with the rpoS mutation repaired using the suicide vector pYA3467 (JSG4383, WT) (57). *S*. Typhi was streaked on Luria-Bertani (LB) agar plates and incubated at 37°C overnight. Single colonies were used to start overnight liquid cultures in tryptic soy broth (TSB) which were grown at 37°C on a rotating drum.

### Murine model of *S*. Typhi carriage

Male and female mice approximately 8–10 weeks old were fed either a lithogenic diet (conventional mouse chow [Teklad LM-485] supplemented with 1% cholesterol and 0.5% cholic acid; Envigo TD.140673) for 6–8 weeks to induce gallstone formation or conventional mouse chow (normal diet). All mice were transitioned to and maintained on a normal diet after the initial diet period and were allowed to rest at least 3 days after diet change prior to infection. For standard infections, mice were IP infected with 200 µL total containing 2 × 10^4^ to 5 × 10^5^ CFUs of WT *S*. Typhi in sterile phosphate-buffered saline (PBS) or 200 µL of sterile PBS. The exact bacterial inoculum quantities were validated by plating serial dilutions of each inoculum to LB agar and enumerating the CFUs present after overnight incubation at 37°C. Mice were weighed daily at the same time each day beginning on the day of infection. Infected mice were euthanized, and the entire gallbladder, liver, and spleen was collected aseptically 21 dpi. Gallbladders, livers, and spleens were homogenized in 0.2, 3–5, and 1 mL, respectively, of sterile PBS using a Fisherbrand 150 tissue homogenizer (Fisher, 15340167). Homogenized tissues were serially diluted in sterile PBS, plated on LB agar, and enumerated after overnight incubation at 37°C.

### Histology and immunohistochemistry of gallbladder and gallstones

Tissues were collected and fixed in 10% neutral buffered formalin. Preparation of tissue was performed by the AWRI Histopathology Core as described previously (58). Serial sections of gallbladder and gallstone (5 µm thick) were mounted on glass slides which were air-dried and then heat-fixed for 30 minutes before rehydration and routine staining with H&E or immunofluorescence using *Salmonella*-specific antibodies. For immunofluorescence, tissues were incubated overnight at 4°C in blocking solution (PBS + 0.5% Triton X-100 + 5% horse serum) containing primary antibodies against *Salmonella* O- and H-antigens (1:500, Novus Biologicals, NB600-1087) and Vi-antigen (1:500, NIBSC, 16/138). Slides were washed three times with PBS + 1% Triton X-100 (PBST), and autofluorescence was quenched with brief incubation in TrueBlack Lipofuscin (Biotium, 23007). Slides were then washed three times in PBST and incubated with fluorescent secondary antibodies in blocking solution for 2 hours at room temperature. After a final wash of three times in PBST, slides were mounted with Fluoromount-G (SouthernBiotech, 0100-01) containing Hoechst 3458 (BD Pharmingen, 565877).

Imaging of H&E stained tissue was conducted using an Olympus BX53 microscope equipped with a 100× (oil) lens and cellSens imaging software. Fluorescent imaging was conducted using a Nikon AX R point scanning confocal microscope equipped with 20× (dry) and 63× (oil) immersion lenses.

### Anti-biofilm compounds

Anti-biofilm compounds JG-1, NDM-35, and NDM-55 were synthesized using techniques previously published (29), which are described in further detail along with NMR data contained within Fig. S2 Supporting Information.

### Treatment of *S*. Typhi-infected CC003/Unc mice with anti-biofilm compounds in conjunction with ciprofloxacin

Mice previously fed lithogenic diet were randomly assigned to one of four groups corresponding to the following treatments: vehicle (10% DMSO, 5% EtOH, 5% Tween-20 in PBS), ciprofloxacin (2 mg/kg/day), NDM-35 (20 mg/kg/day for the first 6 days and 10 mg/kg/day for the final 4 days) + ciprofloxacin (2 mg/kg/day), and NDM-55 (10 mg/kg/day) + ciprofloxacin (2 mg/kg/day). Following infection as described above with 2 × 10^5^ CFU *S*. Typhi, beginning 10 dpi, mice were given daily intraperitoneal (IP) injections of the described compounds or control in a solution of 10% DMSO, 5% EtOH, 5% Tween-20 in sterile PBS for a total volume of 10 µL/gram of mouse. At 21 dpi, mice were euthanized, and the gallbladder, liver, and spleen were collected aseptically. The tissues were homogenized in sterile PBS using a Fisherbrand 150 tissue homogenizer (Fisher, 15340167). Homogenized tissues were serially diluted in sterile PBS, plated on LB agar, and enumerated after overnight incubation at 37°C.

### Biofilm disruption assays, quantification, and EC_50_ determination

Biofilm dispersion was assessed using an *in vitro* biofilm assay, modified from a previously described assay to improve *S*. Typhi biofilm growth (19, 59). *S.* Typhi biofilms were grown in flat-bottom polystyrene 96-wells. To simulate growth on cholesterol gallstones, 96-well plates were pre-coated with cholesterol by adding 100 µL per well of cholesterol solution (5 mg/mL cholesterol dissolved in equal parts isopropanol/ethanol) and incubated overnight at room temperature to allow for evaporation of the solvent. Overnight liquid cultures of *S*. Typhi grown in TSB were normalized to an OD_600_ of 0.8, diluted 1:2,500 in TSB, and added to cholesterol-coated 96-well plates (100 µL/well). Biofilms were incubated at 25°C on a nutating mixer (20° fixed angle, 24 rpm), with media removed and replaced with fresh media every 24 hours.

At 96 hours, the media was replaced with media containing designated concentrations of anti-biofilm compounds or vehicle. Biofilms were incubated with compounds for an additional 24 hours prior to analysis with crystal violet staining. After incubation with compounds, spent media with planktonic cells was removed and wells were washed three times with dH_2_O to remove any bacteria not within the biofilm. The remaining biofilms were then heat-fixed for 1 hour at 60°C prior to staining with crystal violet solution (6 mL PBS, 3.3 mL crystal violet, 333 µL methanol, and 333 µL isopropanol) for 5 minutes. The plates were submerged in dH_2_O to remove unbound crystal violet, and the remaining crystal violet bound biofilms were solubilized with 33% glacial acetic acid. The optical density of the solution was read at 570 nm (OD_570_) using a spectrophotometer (Molecular Devices, SpectraMax M5).

Half-maximal concentrations (EC_50_), defined here as the concentration needed to disperse 50% of the biofilm remaining to vehicle control (DMSO), were calculated for disruption assays using measurements of biofilm remaining after 24 hours in the presence of various concentrations of compounds (6–200 µM).

### Statistical methods

*In vitro* biofilm experiments were performed with three or more technical replicates in each plate and repeated at least three times with biological replicates. Specific statistical analyses are described in the figure legends and were performed using the software GraphPad Prism version 10.3.1.

## SUPPLEMENTAL MATERIAL

Fig. S1 mbio.03476-25-s0001.pdf Liver weights in mice fed 8 weeks of lithogenic diet.

Fig. S2 mbio.03476-25-s0002.pdf Synthesis of compounds NDM-35 and NDM-55.

ASM does not own the copyrights to Supplemental Material that may be linked to, or accessed through, an article. The authors have granted ASM a non-exclusive, world-wide license to publish the Supplemental Material files. Please contact the corresponding author directly for reuse.
