# Supplementary material for: A mouse model for studying chronic Salmonella Typhi infection and anti-biofilm interventions
Source: mBio. 2025 Dec 18;17(2):e03476-25. doi: 10.1128/mbio.03476-25 (PMC12892968; doi:10.1128/mbio.03476-25)
Supplement: Fig. S1 — Liver weights in mice fed 8 weeks of lithogenic diet. [file mbio.03476-25-s0001.pdf]

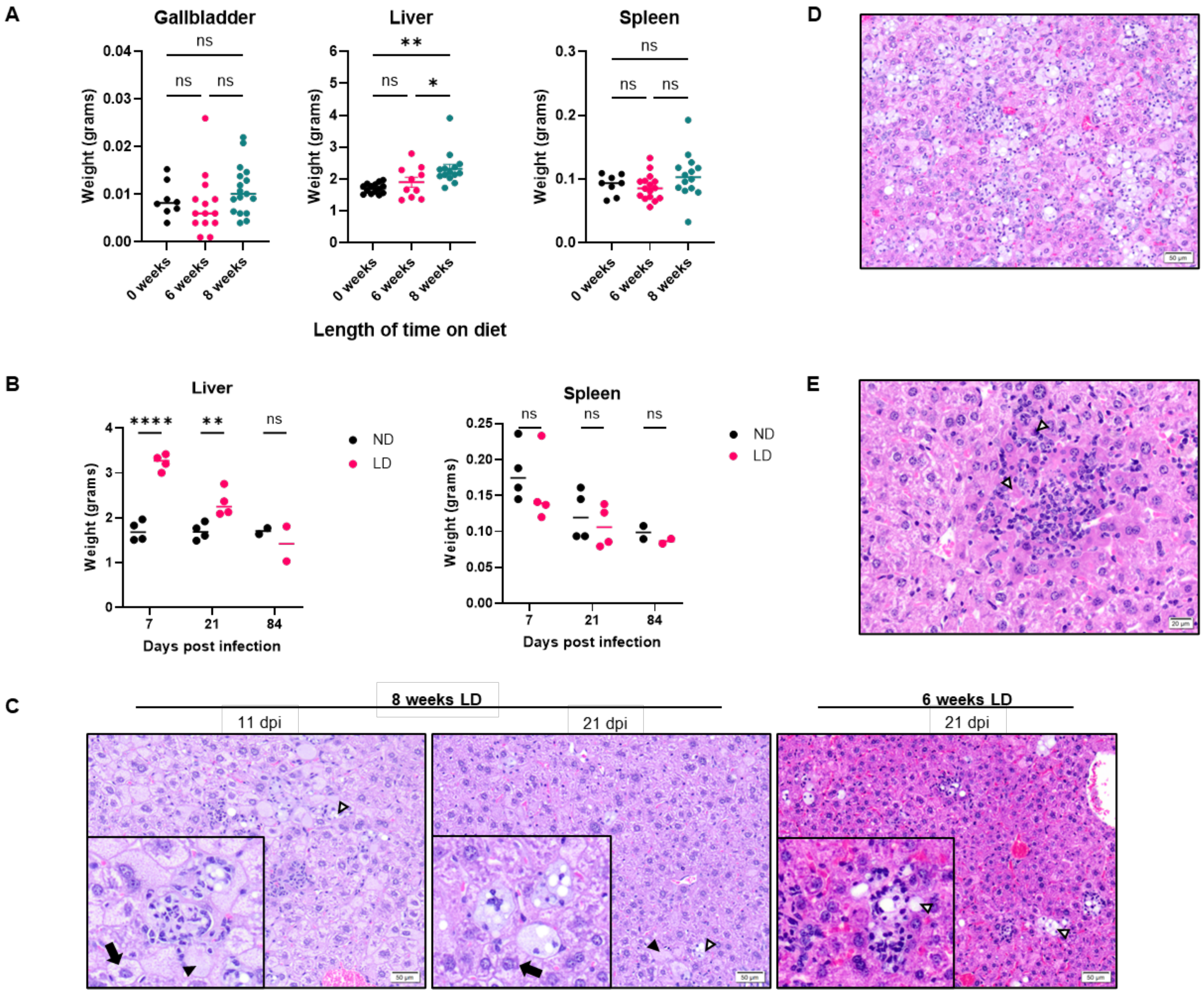

S1 Fig. Liver weights are significantly increased in mice fed 8 weeks of lithogenic diet, which recovers over time after return to normal diet

(A) Mice were fed 6 or 8 weeks of lithogenic diet (LD) or normal diet before IP *S*. Typhi infection followed by tissue collection and organ weight recording at 21 days post infection (dpi). 1-way ANOVA; ** = p-value: 0.0026; * = p-value: 0.0464. (B) Mice were fed 8 weeks of LD or ND before IP *S*. Typhi infection. Mice were returned to normal diet, and tissues were collected and weighed at 7, 21, and 84 dpi. 2-way ANOVA **** = p-value: <0.0001; ** = p-value: 0.0037. (C) Histologic changes in the liver at 11 and 21 dpi. Hepatocytes diffusely have either irregular lacey cytoplasm (glycogen, black arrows) or are filled with numerous small clear vacuoles that rarely displace the nucleus (microvesicular lipid, black arrowheads). Multifocally throughout the liver are

random foci containing variable amounts and ratios of lipid-laden macrophages, neutrophils, and mononuclear cells (lipogranulomas, white arrowheads). 20X. H&E. (D) Histologic changes in the liver of an uninfected CC003 mouse fed a lithogenic diet. (E) Histologic changes in the liver at 21 dpi following protocol adjustments. Throughout the liver are multifocal random foci of mixed inflammatory infiltrates and hepatocytes with hyperosinophilic cytoplasm and occasionally pyknotic, karyolytic, or karyorrhectic nuclei (white arrowheads).
