# Supplementary material for: A mouse model for studying chronic Salmonella Typhi infection and anti-biofilm interventions
Source: mBio. 2025 Dec 18;17(2):e03476-25. doi: 10.1128/mbio.03476-25 (PMC12892968; doi:10.1128/mbio.03476-25)
Supplement: Fig. S2 — Synthesis of compounds NDM-35 and NDM-55. [file mbio.03476-25-s0002.pdf]

S2 Fig. Synthesis of compounds NDM-35 and NDM-55

Chemistry Experimental: All reagents used for chemical synthesis were purchased from commercially available sources (VWR U.S., Fisher Scientific U.S., or Sigma Aldrich U.S.) and used without further purification. Flash chromatography was performed using 60 Å mesh standard grade silica gel from Sorbetch. NMR solvents were obtained from Cambridge Isotope Labs and used as is. All ^{1}H NMR and ^{13}C NMR were recorded at 25˚C on Bruker AVANCE III HD spectrometers (400 MHz). Chemical shifts (δ) are given in parts per million (ppm) relative to the respective NMR solvent; coupling constants (*J*) are in hertz (Hz). Abbreviations used are s, singlet; d, doublet; dd, doublet of doublets; ddd, doublet of doublet of doublets; t, triplet; m, multiplet. High-resolution mass spectrometry measurements were obtained at the Notre Dame Department of Chemistry Mass Spectrometry and Proteomics Facility. Infrared spectra were obtained on a Bruker Alpha II FTIR spectrophotometer (υ_{max} in cm^{-1}). UV absorbance was recorded on a Genesys 10 scanning UV/visible spectrophotometer (λ_{max} in nm). The purities of the tested compounds were all verified to be ≥ 95% by LC-MS analysis on an Advion LC-MS 2020 with Kinetex, 2.6 mm, C_{18} 50 × 2.10 mm, using 20-100% acetonitrile/water with 0.1% formic acid for either 5 or 3 minutes.

General Synthetic Procedure for Piperidine Alkylation. To an oven-dried two-necked 100 mL round bottom flask under argon was added anhydrous acetonitrile (30 mL) and tert-butyl methyl(piperidin-4-ylmethyl)carbamate (0.50 mmol). Oven-dried potassium carbonate (1.5 mmol) was added to the flask in one portion, and the resulting mixture was heated to reflux. Alkyl bromide was added neat in one portion (1.25 mmol) and the reaction mixture was stirred under reflux for 24 hours. The reaction was then cooled and transferred to a single-necked 250 mL round bottom flask to be concentrated under reduced pressure. The crude product was then taken up in in di-H_2O (50 mL) and extracted with chloroform (3x30 mL). The organic layers were combined and washed with brine (30 mL), dried over anhydrous sodium sulfate, filtered, evaporated in vacuo, and purified via flash chromatography using 2% methanol, 2% triethylamine, and 96% chloroform.

General Synthetic Procedure for Boc-Deprotection. Boc-protected intermediate (0.40 mmol) was dissolved in 1 mL dichloromethane. Trifluoroacetic acid (0.2 mL) was added and the reaction was allowed to stir at room temperature open to atmosphere for 1 hour. Methanol (2 mL) was then added and the mixture was evaporated in vacuo to dryness. Addition of ethanol followed by evaporation was repeated four times (or until no further vapors evolved upon addition of solvent). The crude intermediate was dried for 18 hours under high vacuum and used without further purification.

General Synthetic Procedure for Amine Alkylation. Deprotected intermediate (0.40 mmol) was dissolved in 23 mL anhydrous acetonitrile and transferred to an oven-dried 100 mL three-necked flask under argon. Potassium carbonate (1.50 mmol) was added and the mixture was heated to reflux while stirring. Alkyl bromide (0.45 mmol) was dissolved in 2 mL anhydrous acetonitrile and added to the reaction dropwise over one hour. The reaction was checked for completion by TLC after full addition of bromide, then cooled and transferred to a single-necked 250 mL round bottom flask and evaporated in vacuo. The crude product was then taken up in di-H_2O (50 mL) and extracted with chloroform (3x30 mL). The organic layers were combined and washed with brine (30 mL), dried over anhydrous sodium sulfate, filtered, and evaporated under reduced pressure. The crude mixture was purified via flash chromatography using 2% methanol, 2% triethylamine, and 96% chloroform.

General Synthetic Procedure for Formation of HCl Salts. Pure product was dissolved in 1 mL methanol and concentrated hydrochloric acid (0.2 mL) was added. The solvent was evaporated in vacuo. Ethanol addition and subsequent evaporation was repeated six times (until no further fumes evolved upon solvent addition). The resulting solid was dried under vacuum for 24-48 hours to yield the salt.

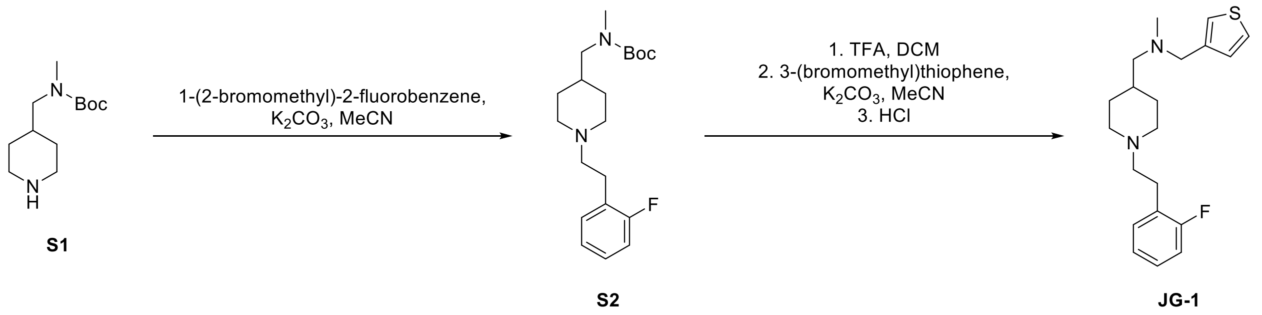

Scheme 1. Synthesis of JG-1

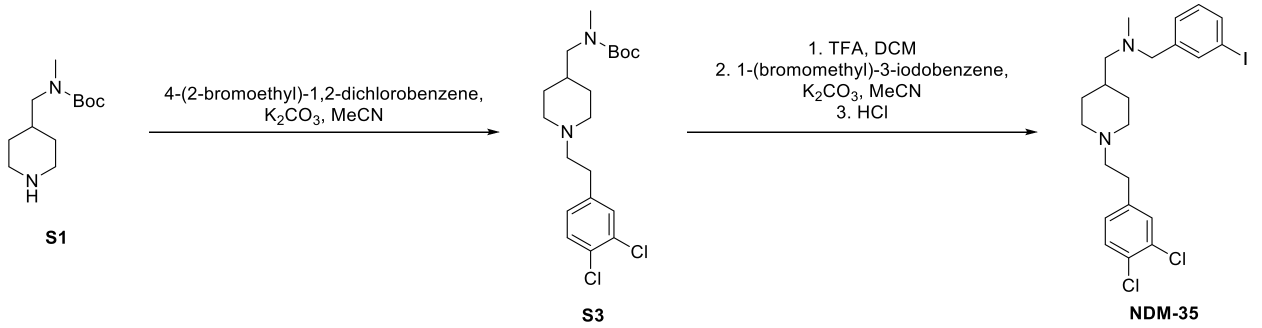

Scheme 2. Synthesis of NDM-35

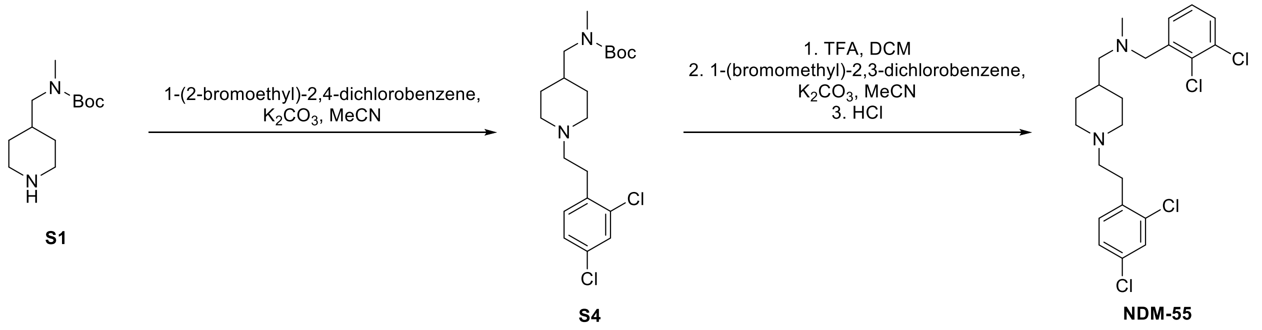

Scheme 3. Synthesis of NDM-55

Previously Reported Compounds

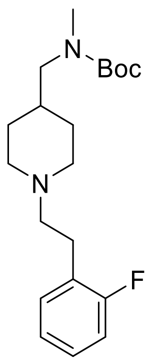

***tert*-Butyl ((1-(2-fluorophenethyl)piperidin-4-yl)methyl)(methyl)carbamate (S2): S1** was reacted with 1-(2-bromoethyl)-2-fluorobenzene and potassium carbonate in acetonitrile following the general procedure for piperidine alkylation.[1] Spectral data was consistent with previous reports.[1]

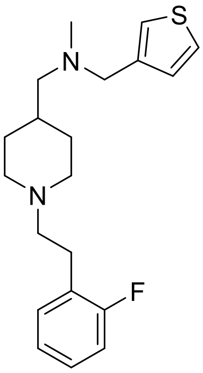

1-(1-(2-Fluorophenethyl)piperidin-4-yl)-*N*-methyl-*N*-(thiophen-3-ylmethyl)methanamine (JG-1): S2 was Boc-deprotected with trifluoroacetic acid in dichloromethane following the general synthetic procedure for Boc-deprotection and subsequently reacted with 3-(bromomethyl)thiophene and potassium carbonate in acetonitrile following the general procedure for amine alkylation.[1] The product was transformed into a salt using the general procedure for making HCl salts.[1] Spectral data was consistent with previous reports.[1]

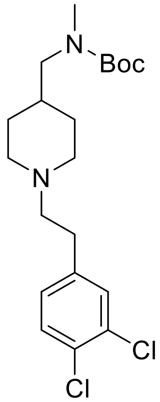

***tert*-Butyl ((1-(3,4-dichlorophenethyl)piperidin-4-yl)methyl)(methyl)carbamate (S3): S1** was reacted with 4-(2-bromoethyl)-1,2-dichlorobenzene and potassium carbonate in acetonitrile following the general procedure for piperidine alkylation.[1] Spectral data was consistent with previous reports.[1]

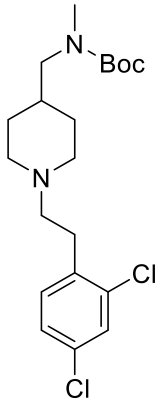

***tert*-Butyl ((1-(2,4-dichlorophenethyl)piperidin-4-yl)methyl)(methyl)carbamate (S4): S1** was reacted with 1-(2-bromoethyl)-2,4-dichlorobenzene and potassium carbonate in acetonitrile following the general procedure for piperidine alkylation.[1] Spectral data was consistent with previous reports.[1]

Novel Compound Characterization

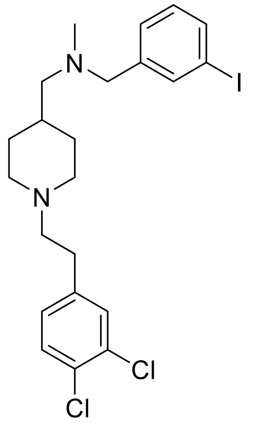

1-(1-(3,4-Dichlorophenethyl)piperidin-4-yl)-*N*-(3-iodobenzyl)-*N*-methylmethanamine

(NDM-35): S3 was Boc-deprotected with trifluoroacetic acid in dichloromethane following the general synthetic procedure for Boc-deprotection and subsequently reacted with 1-(bromomethyl)-3-iodobenzene and potassium carbonate in acetonitrile following the general procedure for amine alkylation. The product was transformed into a salt using the general procedure for making HCl salts and presented as a pale yellow solid (29% yield). ^{1}H NMR (400 MHz, methanol-d$_4$) δ 7.72 (d, *J* = 1.9 Hz, 1H), 7.60 (d, *J* = 7.8 Hz, 1H), 7.49 – 7.39 (m, 2H), 7.31 (d, *J* = 7.9 Hz, 1H), 7.18 (dd, *J* = 8.3, 2.1 Hz, 1H), 7.09 (t, *J* = 7.7 Hz, 1H), 3.45 (s, 2H), 3.18 (d, *J* = 11.6 Hz, 2H), 2.91 – 2.83 (m, 2H), 2.83 – 2.77 (m, 2H), 2.38 (t, *J* = 11.9 Hz, 2H), 2.29 – 2.17 (m, 5H), 1.98 – 1.85 (m, 2H), 1.70 (ddd, *J* = 11.1, 7.3, 3.8 Hz, 1H), 1.34 – 1.19 (m, 2H); ^{13}C NMR (100.6 MHz, methanol-d$_4$) δ 141.7, 139.8, 137.7, 135.9, 131.9, 130.5, 130.3, 130.0, 129.8, 128.4, 128.1, 93.5, 62.6, 61.5, 58.9, 52.9, 32.8, 30.8, 29.3; IR (υ_{max} cm^{-1}) 3435, 2926, 2554, 1470, 1437, 1030, 775; UV (λ_{max} nm) 282; HRMS (ESI) m/z calculated for $C_{22}H_{28}Cl_2IN_2$ $[M+H]^+$: 517.0669, found 517.0661.

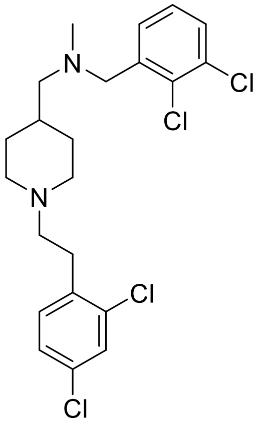

***N*-(2,3-Dichlorobenzyl)-1-(1-(2,4-dichlorophenethyl)piperidin-4-yl)-*N*-methylmethanamine (NDM-55): S4** was Boc-deprotected with trifluoroacetic acid in dichloromethane following the general synthetic procedure for Boc-deprotection and subsequently reacted with 1-(bromomethyl)-2,3-dichlorobenzene and potassium carbonate in acetonitrile following the general procedure for amine alkylation. The product was transformed into a salt using the general procedure for making HCl salts and presented as a pale yellow solid (30% yield). ^{1}H NMR (400 MHz, methanol-d$_4$) δ 7.48 – 7.42 (m, 3H), 7.32 – 7.24 (m, 3H), 3.61 (s, 2H), 3.02 (d, *J* = 11.4 Hz, 2H), 2.98 – 2.87 (m, 2H), 2.62 – 2.52 (m, 2H), 2.30 (d, *J* = 7.2 Hz, 2H), 2.22 (s, 3H), 2.13 (t, *J* = 11.6 Hz, 2H), 1.86 (d, *J* = 13.4 Hz, 2H), 1.66 – 1.54 (m, 1H), 1.27 – 1.15 (m, 2H);^{13}C NMR (100.6 MHz, methanol-d$_4$) δ 139.4, 136.0, 134.3, 132.6, 131.9, 131.8, 129.0, 128.8, 128.7, 127.2, 127.0, 63.4, 59.9, 57.8, 53.0, 41.7, 33.4, 29.8, 29.4; IR (υmax cm^{-1}) 2924, 2792, 1471, 1050, 777; UV (λ_{max} nm) 254; HRMS (ESI) m/z calculated for $C_{22}H_{28}Cl_4N_2$ $[M+H]^+$: 459.0923, found 459.0920.

NMR Spectra

1-(1-(3,4-Dichlorophenethyl)piperidin-4-yl)-*N*-(3-iodobenzyl)-*N*-methylmethanamine

(NDM-35):

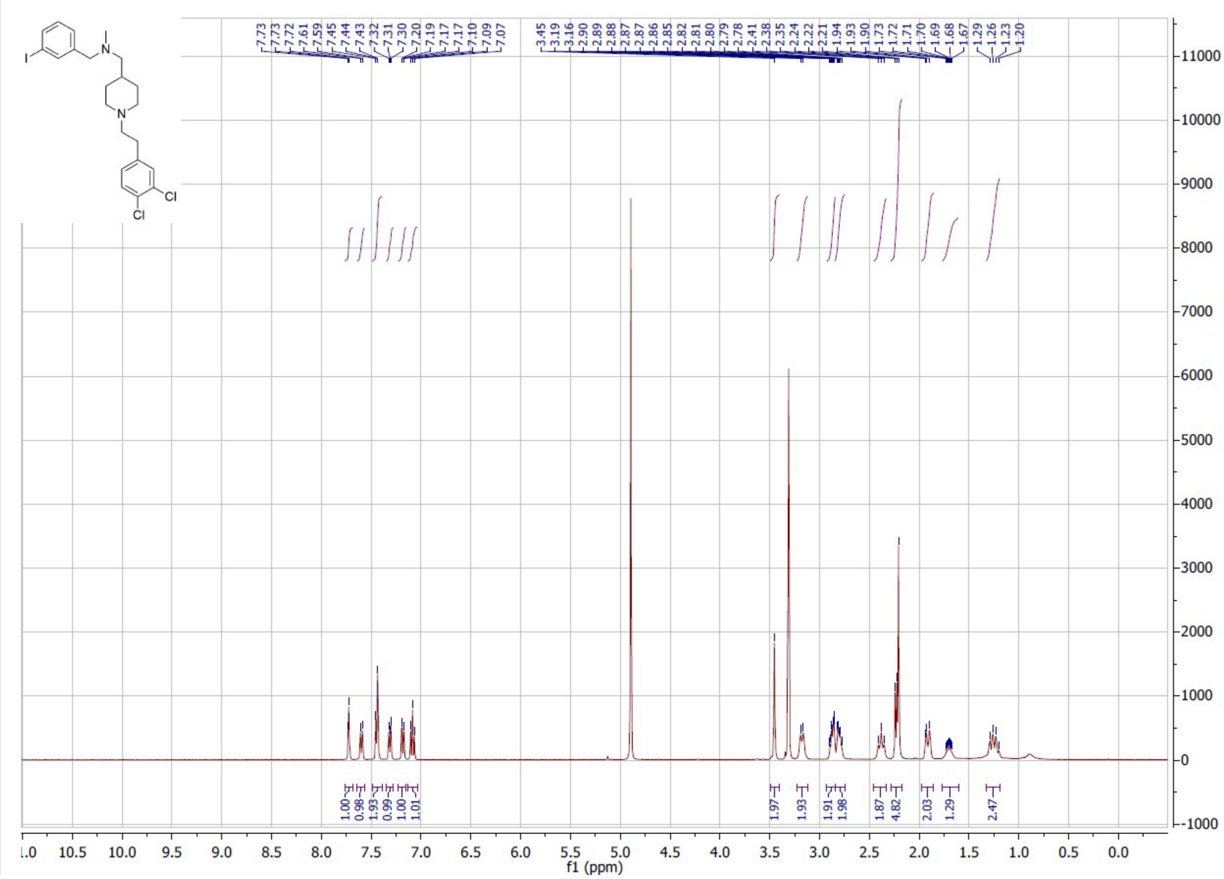

Cl
Cl
I
N
N
f1 (ppm)

N-(2,3-dichlorobenzyl)-1-(1-(2,4-dichlorophenethyl)piperidin-4-yl)-N-methylmethanamine (NDM-55):

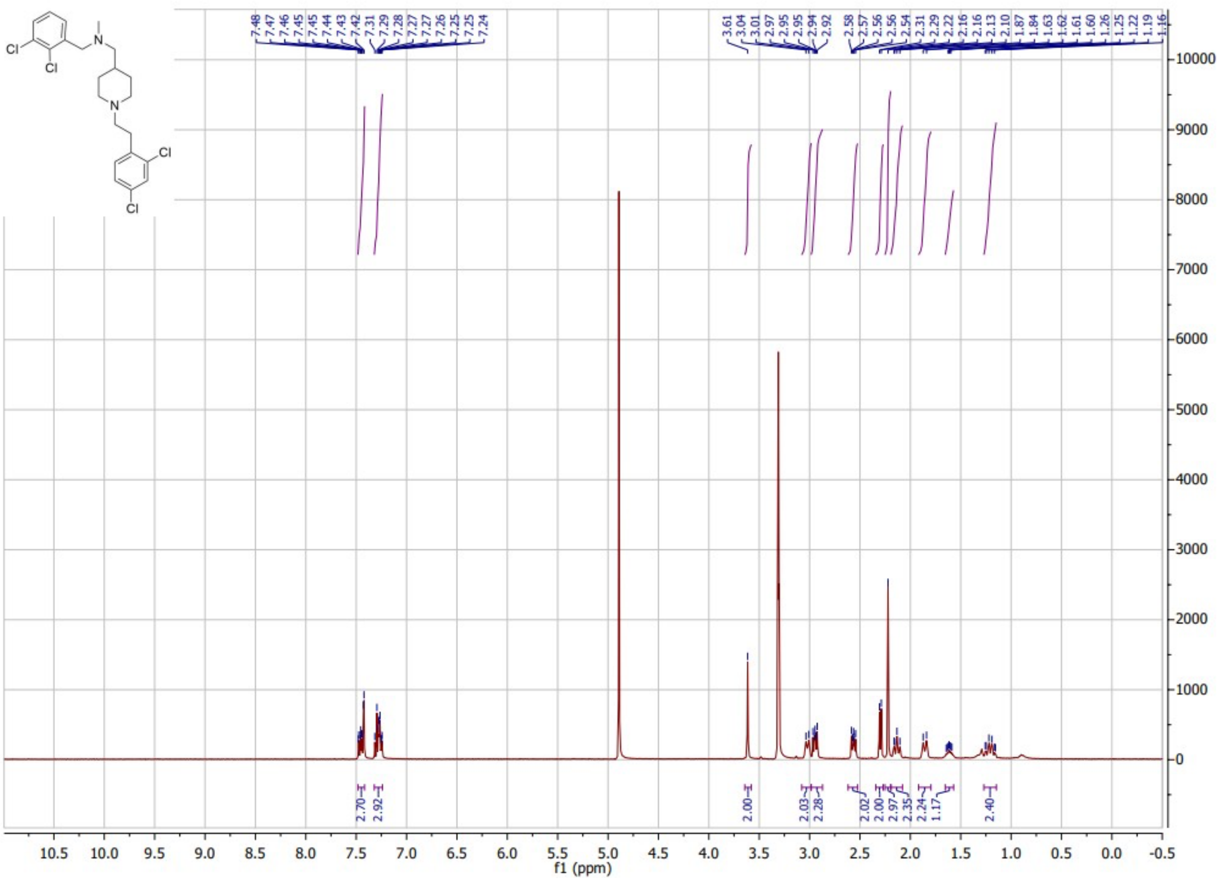

References:

(1) Woolard, K. J.; Sandala, J. L.; Melander, R. J.; Gunn, J. S.; Melander, C. Development of Small Molecules That Work Cooperatively with Ciprofloxacin to Clear Salmonella Biofilms in a Chronic Gallbladder Carriage Model. *European Journal of Medicinal Chemistry* 2022, *232*, 114203. https://doi.org/10.1016/j.ejmech.2022.114203.
